# Supplementary figures and images for: Towards a molecular mechanism underlying mitochondrial protein import through the TOM and TIM23 complexes
Source: eLife. 2022 Jun 8;11:e75426. doi: 10.7554/eLife.75426 (PMC9255969; doi:10.7554/eLife.75426)

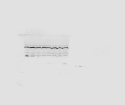

Supplement: Figure 1—figure supplement 1—source data 1. [file elife-75426-fig1-figsupp1-data1.zip › Figure 1 - figure supplement 1 - source data 1/aquisition files/0007769_01_TH.jpg]

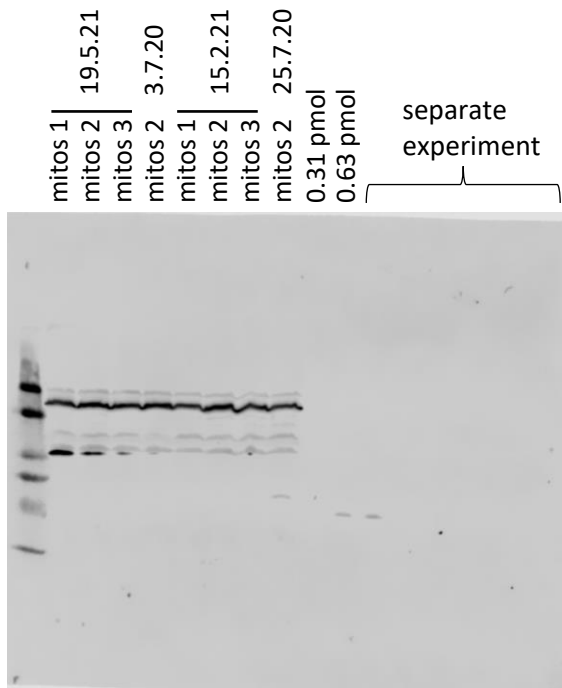

short exposure

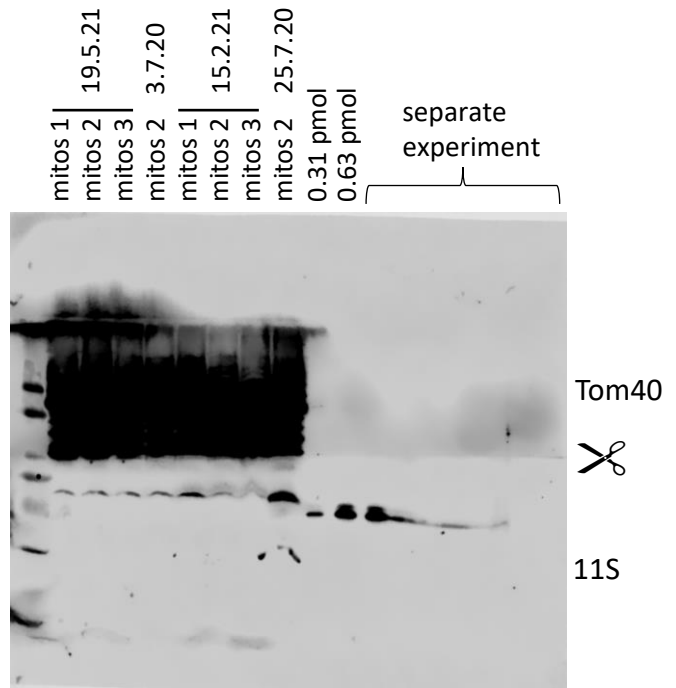

long exposure

Supplement: Figure 1—figure supplement 1—source data 1. [file elife-75426-fig1-figsupp1-data1.zip › Figure 1 - figure supplement 1 - source data 1/Figure 1 - figure supplement 1 - source data 1.pdf]

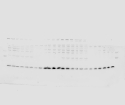

Supplement: Figure 1—figure supplement 1—source data 4. — Image of the Western blot and values from band densitometry used to calculate the proportion of matrix and IMS markers in supernatants following digitonin treatment of mitochondria. Also numerical data corresponding to the amplitudes and luminescence traces from which these were obtained. [file elife-75426-fig1-figsupp1-data4.zip › Figure 1 - figure supplement 1 - source data 4/aquisition files/0007479_01_TH.jpg]

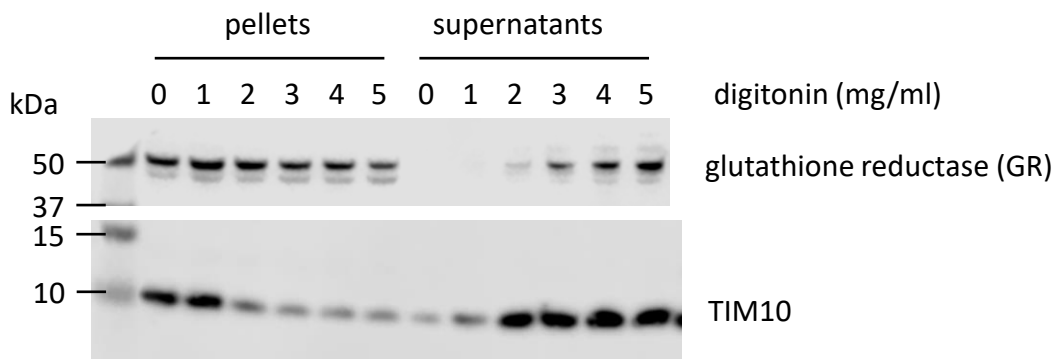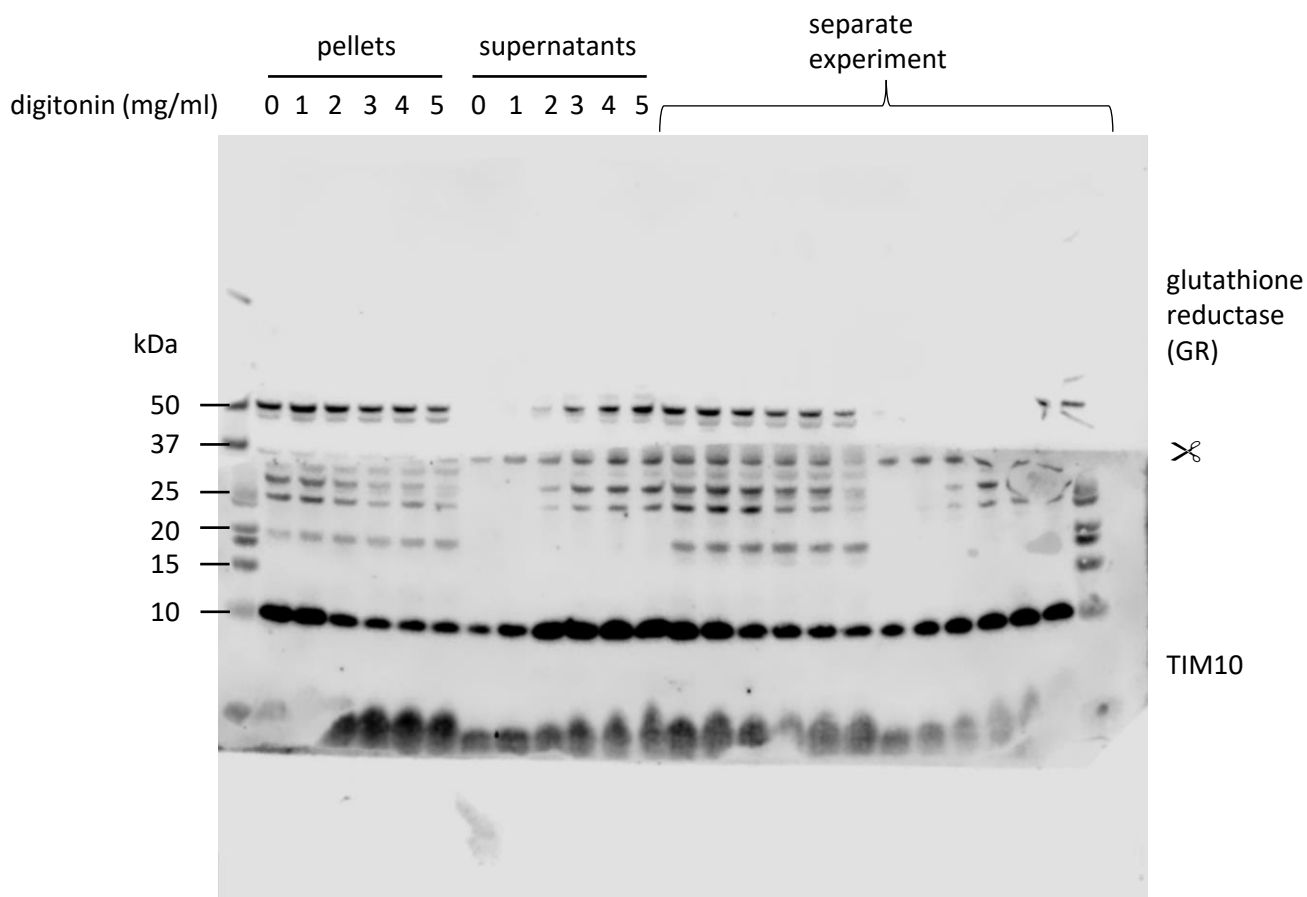

Supplement: Figure 1—figure supplement 1—source data 4. — Image of the Western blot and values from band densitometry used to calculate the proportion of matrix and IMS markers in supernatants following digitonin treatment of mitochondria. Also numerical data corresponding to the amplitudes and luminescence traces from which these were obtained. [file elife-75426-fig1-figsupp1-data4.zip › Figure 1 - figure supplement 1 - source data 4/Figure 1 - figure supplement 1 - source data 4.pdf]

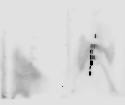

Supplement: Figure 2—figure supplement 1—source data 1. [file elife-75426-fig2-figsupp1-data1.zip › Figure 2 - figure supplement 1 - source data 1/0008147_01_TH.jpg]
