# Supplementary material for: Towards a molecular mechanism underlying mitochondrial protein import through the TOM and TIM23 complexes
Source: eLife. 2022 Jun 8;11:e75426. doi: 10.7554/eLife.75426 (PMC9255969; doi:10.7554/eLife.75426)
Supplement: Supplementary file 1. [file elife-75426-supp1.docx]

| his tag  myc tag  v5 tag  pep86 “L”  scrambled pep86 “D”  *S. cerevisiae* mature Acp1  *S. cerevisiae* Acp1 presequence  *M. musculus* DHFR  *N. crassa* Su9 1-69  *S. cerevisiae* cyt *b_2_* 1-191 ∆43-65 | | |
| --- | --- | --- |
| **PCP name** | **Amino acid sequence** | **Expression vector** |
| PCP-DHFR-pep86 | MASTRVLASRLASQMAASAKVARPAVRVAQVSKRTIQTGSPLQTLKRTQMTSIVNATTRQAFQKRAYSSSANLSKDQVSQRVIDVIKAFDKNSPNIANKQISSDTQFHKDLGLDSLDTVELLVAIEEEFDIEIPDKVADELRSVGETVDYIASNPDANGSGVSWGLRKFKISGSGSANLSKDQVSQRVIDVIKAFDKNSPNIANKQISSDTQFHKDLGLDSLDTVELLVAIEEEFDIEIPDKVADELRSVGETVDYIASNPDANGSGVSWGLRKFKISVRPLNSIVAVSQNMGIGKNGDLPWPPLRNEFKYFQRMTTTSSVEGKQNLVIMGRKTWFSIPEKNRPLKDRINIVLSRELKEPPRGAHFLAKSLDDALRLIEQPELASKVDMVWIVGGSSVYQEAMNQPGHLRLFVTRIMQEFESDTFFPEIDLGKYKLLPEYPGVLSEVQEEKGIKYKFEVYEKKDFEAYVEQKLISEEDLNSAVVSGWRLFKKIS | pE-SUMOpro |
| PCP-pep86-DHFR | MASTRVLASRLASQMAASAKVARPAVRVAQVSKRTIQTGSPLQTLKRTQMTSIVNATTRQAFQKRAYSSSANLSKDQVSQRVIDVIKAFDKNSPNIANKQISSDTQFHKDLGLDSLDTVELLVAIEEEFDIEIPDKVADELRSVGETVDYIASNPDANGSGVSGWRLFKKISGSGSANLSKDQVSQRVIDVIKAFDKNSPNIANKQISSDTQFHKDLGLDSLDTVELLVAIEEEFDIEIPDKVADELRSVGETVDYIASNPDANGSGVSWGLRKFKISVRPLNSIVAVSQNMGIGKNGDLPWPPLRNEFKYFQRMTTTSSVEGKQNLVIMGRKTWFSIPEKNRPLKDRINIVLSRELKEPPRGAHFLAKSLDDALRLIEQPELASKVDMVWIVGGSSVYQEAMNQPGHLRLFVTRIMQEFESDTFFPEIDLGKYKLLPEYPGVLSEVQEEKGIKYKFEVYEKKDFEAYVEQKLISEEDLNSAVC | pE-SUMOpro |
| L (also PCP-pep86) | MVFRSVCRISSRVAPSAYRTIMGRSVMSNTILAQRFYSANLSKDQVSQRVIDVIKAFDKNSPNIANKQISSDTQFHKDLGLDSLDTVELLVAIEEEFDIEIPDKVADELRSVGETVDYIASNPDANGSGVSGWRLFKKISGSGEQKLISEEDLGGHHHHHH | pRSFDuet |
| DL | MVFRSVCRISSRVAPSAYRTIMGRSVMSNTILAQRFYSANLSKDQVSQRVIDVIKAFDKNSPNIANKQISSDTQFHKDLGLDSLDTVELLVAIEEEFDIEIPDKVADELRSVGETVDYIASNPDANGSGVSWGLRKFKISGSGSANLSKDQVSQRVIDVIKAFDKNSPNIANKQISSDTQFHKDLGLDSLDTVELLVAIEEEFDIEIPDKVADELRSVGETVDYIASNPDANGSGVSGWRLFKKISGSGEQKLISEEDLGGHHHHHH | pRSFDuet |
| DDL | MVFRSVCRISSRVAPSAYRTIMGRSVMSNTILAQRFYSANLSKDQVSQRVIDVIKAFDKNSPNIANKQISSDTQFHKDLGLDSLDTVELLVAIEEEFDIEIPDKVADELRSVGETVDYIASNPDANGSGVSWGLRKFKISGSGSANLSKDQVSQRVIDVIKAFDKNSPNIANKQISSDTQFHKDLGLDSLDTVELLVAIEEEFDIEIPDKVADELRSVGETVDYIASNPDANGSGVSWGLRKFKISGSGSANLSKDQVSQRVIDVIKAFDKNSPNIANKQISSDTQFHKDLGLDSLDTVELLVAIEEEFDIEIPDKVADELRSVGETVDYIASNPDANGSGVSGWRLFKKISGSGEQKLISEEDLGGHHHHHH | pRSFDuet |
| DDDL | MVFRSVCRISSRVAPSAYRTIMGRSVMSNTILAQRFYSANLSKDQVSQRVIDVIKAFDKNSPNIANKQISSDTQFHKDLGLDSLDTVELLVAIEEEFDIEIPDKVADELRSVGETVDYIASNPDANGSGVSWGLRKFKISGSGSANLSKDQVSQRVIDVIKAFDKNSPNIANKQISSDTQFHKDLGLDSLDTVELLVAIEEEFDIEIPDKVADELRSVGETVDYIASNPDANGSGVSWGLRKFKISGSGSANLSKDQVSQRVIDVIKAFDKNSPNIANKQISSDTQFHKDLGLDSLDTVELLVAIEEEFDIEIPDKVADELRSVGETVDYIASNPDANGSGVSWGLRKFKISGSGSANLSKDQVSQRVIDVIKAFDKNSPNIANKQISSDTQFHKDLGLDSLDTVELLVAIEEEFDIEIPDKVADELRSVGETVDYIASNPDANGSGVSGWRLFKKISGSGEQKLISEEDLGGHHHHHH | pRSFDuet |
| LDDD | MVFRSVCRISSRVAPSAYRTIMGRSVMSNTILAQRFYSANLSKDQVSQRVIDVIKAFDKNSPNIANKQISSDTQFHKDLGLDSLDTVELLVAIEEEFDIEIPDKVADELRSVGETVDYIASNPDANGSGVSGWRLFKKISGSGSANLSKDQVSQRVIDVIKAFDKNSPNIANKQISSDTQFHKDLGLDSLDTVELLVAIEEEFDIEIPDKVADELRSVGETVDYIASNPDANGSGVSWGLRKFKISGSGSANLSKDQVSQRVIDVIKAFDKNSPNIANKQISSDTQFHKDLGLDSLDTVELLVAIEEEFDIEIPDKVADELRSVGETVDYIASNPDANGSGVSWGLRKFKISGSGSANLSKDQVSQRVIDVIKAFDKNSPNIANKQISSDTQFHKDLGLDSLDTVELLVAIEEEFDIEIPDKVADELRSVGETVDYIASNPDANGSGVSWGLRKFKISGSGEQKLISEEDLGGHHHHHH | pRSFDuet |
| DLDD | MVFRSVCRISSRVAPSAYRTIMGRSVMSNTILAQRFYSANLSKDQVSQRVIDVIKAFDKNSPNIANKQISSDTQFHKDLGLDSLDTVELLVAIEEEFDIEIPDKVADELRSVGETVDYIASNPDANGSGVSWGLRKFKISGSGSANLSKDQVSQRVIDVIKAFDKNSPNIANKQISSDTQFHKDLGLDSLDTVELLVAIEEEFDIEIPDKVADELRSVGETVDYIASNPDANGSGVSGWRLFKKISGSGSANLSKDQVSQRVIDVIKAFDKNSPNIANKQISSDTQFHKDLGLDSLDTVELLVAIEEEFDIEIPDKVADELRSVGETVDYIASNPDANGSGVSWGLRKFKISGSGSANLSKDQVSQRVIDVIKAFDKNSPNIANKQISSDTQFHKDLGLDSLDTVELLVAIEEEFDIEIPDKVADELRSVGETVDYIASNPDANGSGVSWGLRKFKISGSGEQKLISEEDLGGHHHHHH | pRSFDuet |
| DDLD | MVFRSVCRISSRVAPSAYRTIMGRSVMSNTILAQRFYSANLSKDQVSQRVIDVIKAFDKNSPNIANKQISSDTQFHKDLGLDSLDTVELLVAIEEEFDIEIPDKVADELRSVGETVDYIASNPDANGSGVSWGLRKFKISGSGSANLSKDQVSQRVIDVIKAFDKNSPNIANKQISSDTQFHKDLGLDSLDTVELLVAIEEEFDIEIPDKVADELRSVGETVDYIASNPDANGSGVSWGLRKFKISGSGSANLSKDQVSQRVIDVIKAFDKNSPNIANKQISSDTQFHKDLGLDSLDTVELLVAIEEEFDIEIPDKVADELRSVGETVDYIASNPDANGSGVSGWRLFKKISGSGSANLSKDQVSQRVIDVIKAFDKNSPNIANKQISSDTQFHKDLGLDSLDTVELLVAIEEEFDIEIPDKVADELRSVGETVDYIASNPDANGSGVSWGLRKFKISGSGEQKLISEEDLGGHHHHHH | pRSFDuet |
| -13pos | MVKYKPLLKISKNSEAAILRASKTRLNTIRAYGSTVPKSKSFSSVAYLNWHNGQIDNEPQLDMNQGGIPNPLLGLGGPAEVAQHNQPDDCWVVINGYVYDLTQFLPNHPGGQDVIQFNAGQDVTAIFEPLHAPNVIDQYIAPEQQLGPLQGSMPPELVCPPYAPGETQEDIAQQEQGTLQHHHHHHSGGGGSVSGWRLFKKIS | pBAD |
| -8pos | MVKYKPLLKISKNSEAAILRASKTRLNTIRAYGSTVPKSKSFSSVAYLNWHNGQIDNEPQLDMNQGGIPNPLLGLGGPAEVAQHNKPDDCWVVINGYVYDLTQFLPNHPGGQDVIQFNAGKDVTAIFEPLHAPNVIDQYIAPEKKLGPLQGSMPPELVCPPYAPGETQEDIAQKEQGTLQHHHHHHSGGGGSVSGWRLFKKIS | pRSFDuet |
| +8neg | MVKYKPLLKISKNSEAAILRASKTRLNTIRAYGSTVPKSKSFSSVAYLNWHNGQIDNEPKLDMDKGGIPNPLLGLGGPAEVAKHDKPDDCWVVIDGYVYDLTRFLPDHPGGQDVIKFDAGKDVTAIFEPLHAPDVIDKYIAPEKKLGPLEGSMPPELVCPPYAPGETKEDIARKEEGTLQHHHHHHSGGGGSVSGWRLFKKIS | pRSFDuet |
| Native | MVKYKPLLKISKNSEAAILRASKTRLNTIRAYGSTVPKSKSFSSVAYLNWHNGQIDNEPKLDMNKGGIPNPLLGLGGPAEVAKHNKPDDCWVVINGYVYDLTRFLPNHPGGQDVIKFNAGKDVTAIFEPLHAPNVIDKYIAPEKKLGPLQGSMPPELVCPPYAPGETKEDIARKEQGTLQHHHHHHSGGGGSVSGWRLFKKIS | pBAD |
| +8pos | MVKYKPLLKISKNSEAAILRASKTRLNTIRAYGSTVPKSKSFSSVAYLNWHNGQIDNEPKLDMKKGGIPNPLLGLGGPAEVAKHNKPDDCWVVIKGYVYDLTRFLPKHPGGRDVIKFKAGKDVTAIFEPLHAPKVIDKYIAPEKKLGPLRGSMPPELVCPPYAPGETKEDIARKERGTLQHHHHHHSGGGGSVSGWRLFKKIS | pRSFDuet |
| -8neg | MVKYKPLLKISKNSEAAILRASKTRLNTIRAYGSTVPKSKSFSSVAYLNWHNGQIDNEPKLNMNKGGIPNPLLGLGGPAQVAKHNKPDDCWVVINGYVYNLTRFLPNHPGGQNVIKFNAGKDVTAIFQPLHAPNVIDKYIAPQKKLGPLQGSMPPQLVCPPYAPGQTKEDIARKEQGTLQHHHHHHSGGGGSVSGWRLFKKIS | pBAD |
| -17neg | MVKYKPLLKISKNSEAAILRASKTRLNTIRAYGSTVPKSKSFSSVAYLNWHNGQINNQPKLNMNKGGIPNPLLGLGGPAQVAKHNKPNNCWVVINGYVYNLTRFLPNHPGGQNVIKFNAGKNVTAIFQPLHAPNVINKYIAPQKKLGPLQGSMPPQLVCPPYAPGQTKQNIARKQQGTLQHHHHHHSGGGGSVSGWRLFKKIS | pBAD |
| F_1_ α | MVLARTAAIRSLSRTLINSTKAARPAAAALASTRRLASTKAQPTEVSSILEERIKGVSDEANLNETGRVLAVGDGIARVFGLNNIQAEELVEFSSGVKGMALNLEPGQVGIVLFGSDRLVKEGELVKRTGNIVDVPVGPGLLGRVVDALGNPIDGKGPIDAAGRSRAQVKAPGILPRRSVHEPVQTGLKAVDALVPIGRGQRELIIGDRQTGKTAVALDTILNQKRWNNGSDESKKLYCVYVAVGQKRSTVAQLVQTLEQHDAMKYSIIVAATASEAAPLQYLAPFTAASIGEWFRDNGKHALIVYDDLSKQAVAYRQLSLLLRRPPGREAYPGDVFYLHSRLLERAAKLSEKEGSGSLTALPVIETQGGDVSAYIPTNVISITDGQIFLEAELFYKGIRPAINVGLSVSRVGSAAQVKALKQVAGSLKLFLAQYREVAAFAQFGSDLDASTKQTLVRGERLTQLLKQNQYSPLATEEQVPLIYAGVNGHLDGIELSRIGEFESSFLSYLKSNHNELLTEIREKGELSKELLASLKSATESFVATFGGEQKLISEEDLGGHHHHHHGGVSGWRLFKKIS | pBAD |
| F_1_ β | MVLPRLYTATSRAAFKAAKQSAPLLSTSWKRCMASAAQSTPITGKVTAVIGAIVDVHFEQSELPAILNALEIKTPQGKLVLEVAQHLGENTVRTIAMDGTEGLVRGEKVLDTGGPISVPVGRETLGRIINVIGEPIDERGPIKSKLRKPIHADPPSFAEQSTSAEILETGIKVVDLLAPYARGGKIGLFGGAGVGKTVFIQELINNIAKAHGGFSVFTGVGERTREGNDLYREMKETGVINLEGESKVALVFGQMNEPPGARARVALTGLTIAEYFRDEEGQDVLLFIDNIFRFTQAGSEVSALLGRIPSAVGYQPTLATDMGLLQERITTTKKGSVTSVQAVYVPADDLTDPAPATTFAHLDATTVLSRGISELGIYPAVDPLDSKSRLLDAAVVGQEHYDVASKVQETLQTYKSLQDIIAILGMDELSEQDKLTVERARKIQRFLSQPFAVAEVFTGIPGKLVRLKDTVASFKAVLEGKYDNIPEHAFYMVGGIEDVVAKAEKLAAEANGGEQKLISEEDLGGHHHHHHGGVSGWRLFKKIS | pBAD |
| Mrp21 | MVLKSTLRLSRISLRRGFTTIDCLRQQNSDIDKIILNPIKLAQGSNSDRGQTSKSKTDNADILSMEIPVDMMQSAGRINKRELLSEAEIARSSVENAQMRFNSGKSIIVNKNNPAESFKRLNRIMFENNIPGDKRSQRFYMKPGKVAELKRSQRHRKEFMMGFKRLIEIVKDAKRKGYEQKLISEEDLGGHHHHHHSGGGGSVSGWRLFKKIS | pBAD |
| Acp1 | MVFRSVCRISSRVAPSAYRTIMGRSVMSNTILAQRFYSANLSKDQVSQRVIDVIKAFDKNSPNIANKQISSDTQFHKDLGLDSLDTVELLVAIEEEFDIEIPDKVADELRSVGETVDYIASNPDANEQKLISEEDLGGHHHHHHSGGGGSVSGWRLFKKIS | pBAD |
